# Proteome readjustments in the apoplastic space of *Arabidopsis thaliana ggt1* mutant leaves exposed to UV-B radiation

**DOI:** 10.3389/fpls.2015.00128

**Published:** 2015-03-24

**Authors:** Anna Rita Trentin, Micaela Pivato, Syed M. M. Mehdi, Leonard Ebinezer Barnabas, Sabrina Giaretta, Marta Fabrega-Prats, Dinesh Prasad, Giorgio Arrigoni, Antonio Masi

**Affiliations:** ^1^Department of Agronomy, Food, Natural Resources, Animals and the Environment, University of PadovaPadova, Italy; ^2^Proteomics Center of Padova UniversityPadova, Italy; ^3^Sugarcane Breeding InstituteCoimbatore, India; ^4^Department of Bio-Engineering, Birla Institute of TechnologyRanchi, India; ^5^Department of Biomedical Sciences, University of PadovaPadova, Italy

**Keywords:** glutathione, gamma-glutamyl-transferase, oxidative stress, iTRAQ labeling, apoplast, ultraviolet-B radiation

## Abstract

Ultraviolet-B radiation acts as an environmental stimulus, but in high doses it has detrimental effects on plant metabolism. Plasma membranes represent a major target for Reactive Oxygen Species (ROS) generated by this harmful radiation. Oxidative reactions occurring in the apoplastic space are counteracted by antioxidative systems mainly involving ascorbate and, to some extent, glutathione. The occurrence of the latter and its exact role in the extracellular space are not well documented, however. In *Arabidopsis thaliana*, the gamma-glutamyl transferase isoform (GGT1) bound to the cell wall takes part in the so-called gamma-glutamyl cycle for extracellular glutathione degradation and recovery, and may be implicated in redox sensing and balance. In this work, oxidative conditions were imposed with Ultraviolet-B radiation (UV-B) and studied in redox altered *ggt1* mutants. The response of *ggt1* knockout *Arabidopsis* leaves to UV-B radiation was assessed by investigating changes in extracellular glutathione and ascorbate content and their redox state, and in apoplastic protein composition. Our results show that, on UV-B exposure, soluble antioxidants respond to the oxidative conditions in both genotypes. Rearrangements occur in their apoplastic protein composition, suggesting an involvement of Hydrogen Peroxide (H_2_O_2_), which may ultimately act as a signal. Other important changes relating to hormonal effects, cell wall remodeling, and redox activities are discussed. We argue that oxidative stress conditions imposed by UV-B and disruption of the gamma-glutamyl cycle result in similar stress-induced responses, to some degree at least. Data are available via ProteomeXchange with identifier PXD001807.

## Introduction

The apoplast—i.e., the extraprotoplastic matrix of plant cells, including the cell wall—contains a number of enzymatic and non-enzymatic components involved in many physiological processes and is therefore important in the plant cell's response to both abiotic and biotic stress (Dietz, 1997; Agrawal et al., 2010). Being at the interface with the external environment, rapid fluctuations occur in this compartment as a consequence of unfavorable conditions, such as salinity (Hernandez et al., 2001), ozone (Jaspers et al., 2005) drought (Hu et al., 2005), and UV-B radiation (Pristov et al., 2013), with consequent changes in the concentrations and redox state of its components.

Ultraviolet-B radiation (UV-B, 280–315 nm) is a component of the solar electromagnetic spectrum reaching the Earth's surface, which has gained attention in recent years because it has increased as a consequence of ozone layer destruction by anthropogenic emissions.

As a component of the solar radiation reaching the leaf, UV-B also acts as an environmental stimulus for plant growth and development. Recent literature has demonstrated the existence of the UV-B photoreceptor 8 (UVR8), which controls the plant's photomorphogenic response to UV-B radiation. UVR8 promotes a signal cascade that mediates UV-B photomorphogenic responses in order to secure plant acclimation and survival in sunlight (Rizzini et al., 2011).

While it is beneficial at low intensities (Hideg et al., 2013), numerous studies have reported that excess UV-B radiation harms plants by causing oxidative damage to cellular targets (Brosché and Strid, 2003), altering the structure and functions of the leaf epidermis, cell wall, and membranes (Pristov et al., 2013). A common consequence of many types of environmental stress in plants is a greater abundance of some reactive oxygen species (ROS), such as superoxide, hydrogen peroxide (H_2_O_2_), hydroxyl radicals and singlet oxygen (Li and van Staden, 1998). Increases in ROS are seen after UV-B exposure too (Noctor et al., 2014), and result in lipid peroxidations and damage to plasma membranes. To prevent these detrimental effects, plant cells deploy an array of non-enzymatic and enzymatic antioxidant systems that act as biochemical barriers to counteract and deactivate ROS.

This complex interplay of several metabolites, enzymes, ROS, antioxidants, and hormones gives rise to signals that are transferred inside the cell through the plasma membrane to activate adaptive and response mechanisms.

A major line of defense in the apoplast is represented by the antioxidant molecule ascorbate and, to a lesser extent, glutathione. While both are involved intracellularly in the Halliwell-Asada pathway for controlling ROS and thereby maintaining the cellular redox state and protecting the cellular components from oxidative threat (Smirnoff and Pallanca, 1996; Schafer and Buettner, 2001; Saruhan et al., 2009; Potters et al., 2010), only ascorbate occurs in high micromolar, or even millimolar quantities in the apoplast (Potters et al., 2010), where it can play a part in redox control. The role of extracellular glutathione in the apoplastic space is controversial because it can only be found in traces under physiological conditions, but it can rise to 2% of the total leaf glutathione under pathogen attack (Vanacker et al., 1998a).

There have been reports, however, of the extracellular enzyme gamma-glutamyl-transferase (GGT; E.C. 2.3.2.2) degrading Glutathione (GSH) (Martin et al., 2007), which means that, like animals (Meister and Anderson, 1983), plants also have a gamma-glutamyl cycle involving intracellular glutathione biosynthesis, extrusion and extracellular degradation, with recovery of the constituent amino acids (Ferretti et al., 2009).

These findings can explain the low levels of glutathione in the extracellular environment on the one hand, but also raise the question of the significance of a gamma-glutamyl cycle in plants. In barley roots, using GGT inhibitors in association with the thiol oxidizing molecule diamide resulted in a net glutathione extrusion and accumulation in the extracellular medium (Ferretti et al., 2009). This leads us to wonder whether a gamma-glutamyl cycle could operate as a redox sensing or redox balancing system.

Another study (Tolin et al., 2013) characterized the leaf proteome of *Arabidopsis thaliana ggt1* mutant lines and showed that, even under physiological conditions, a number of antioxidant and defense enzymes were significantly upregulated as a result of impaired extracellular GGT activity. This also implies that GSH turnover involving apoplastic GSH degradation is needed for proper redox sensing and/or a coordinated response to the environment. We speculated that a feedback signal might be missing when the GGT cycle is disrupted, and this would trigger the altered response.

To shed light on these unknown GGT functions in the plant's adaptation to the environment, in this work we investigated the effects of UV-B radiation as an oxidizing stress condition affecting the apoplastic environment in wild type *Arabidopsis* and a previously-characterized *ggt1* knockout mutant line (Destro et al., 2011).

To improve our understanding of protein regulation, it can be helpful to use fractionation (sub-cellular proteomics) to reduce the complexity of the total protein extract and enable the visualization of proteins occurring in low quantities (Brunet et al., 2003).

Since apoplastic proteome analysis can afford a better understanding of the complex network of extracellular proteins involved in plant defense (Agrawal et al., 2010), we investigated the changes occurring in the extracellular proteome as a consequence of the null mutation and/or UV-B treatment by means of Isobaric tags for relative and absolute quantification (iTRAQ) labeling for relative peptide quantification and Liquid Chromatography Mass Spectrometry (LC-MS-MS) analysis. This strategy enables an accurate and sensitive protein quantification, which is essential for the identification of apoplastic proteins in small quantities or small variations in their level of expression.

Following extraction with the extracellular washing fluid (ECWF) technique, we also explored ascorbate and glutathione content and their redox state in the leaf apoplastic fluids.

## Materials and methods

### Plant materials and growth conditions

Seeds of *A. thaliana* and a *ggt1* knockout mutant line, both Columbia ecotype (Col-0), were sterilized and incubated at 4°C in the dark for 4 days to synchronize germination and ensure a uniform growth. The *ggt1* knockout mutant was established in the mutant collection identified by the Salk Institute (Alonso et al., 2003), and was obtained from the Nottingham *A. thaliana* Stock Centre (http://nasc.nott.ac.uk; polymorphism SALK_080363). Seeds were sown in soil pots and grown in a greenhouse.

For the UV-B radiation experiments, plants in the phase of maximum expansion of the rosette (before bolting) were transferred to a climatic cell 2 days before the treatment to enable their acclimation. The growth chamber settings were: 12/12 h light/dark cycle, 21/21°C temperature, 300 μmol m^−2^ s^−1^ photosynthetically active radiation, and 60% relative humidity. The UV-B treatment was applied for 8 h at the beginning of the light period. The radiation was provided by two Philips TL40W/12 lamps with an intensity, measured on a level with the plants, of 8.3 kJ m^−2^ d^−1^ (UVB_BE_, biologically effective UV-B). After the 8 h UV-B treatment, leaves were immediately harvested for ECWF and total leaf extraction. Following, both the infiltrate and the leaf extracts were analyzed for ascorbate content by spectrophotometric method, as described, the same day. Aliquots of the extracts were stored in −80° for thiol measurements.

### Apoplastic fluid extraction

ECWF were extracted by vacuum infiltration according to Lohaus et al. (2001). About 1 g of fresh leaves were cut, rinsed, immersed in infiltration buffer and vacuum-infiltrated for 10 min at 20 kPa. After infiltration, the leaves were blot-dried, weighed and placed vertically in a 5 ml syringe. The syringes were placed in tubes and centrifuged at 200 g, 4°C for 20 min. Apoplastic fluids were collected from the bottom of the tubes. For ascorbate and thiol extraction, 10 μl 0.1N HCl were placed at the bottom of the tubes before centrifugation to prevent oxidation. The composition of the infiltration buffer used for the ascorbate and thiol measurements was: KH_2_PO_4_ 50 mM, KCl 50 mM, and EDTA 2.5 mM, pH 4.5. For the GGT activity and proteomic analyses, the infiltration buffer contained: KH_2_PO_4_ 50 mM, KCl 0.2 M, and PMSF 1 mM, pH 6.2.

The contamination level of the extracts obtained with the infiltration/centrifugation technique was assessed by means of malic dehydrogenase activity measurements, and ranged between 1.6 and 2.5% among the replicate extractions (data not shown).

### Total leaf extraction

Total leaf extraction for the thiol, ascorbate and Dehydroascorbate (DHA) measurements was done using metaphosphoric acid 1.5% and EDTA 1 mM buffer: 1 g of fresh leaves were powdered in a mortar with liquid nitrogen and extracted in a leaves to buffer ratio of 1:4, then centrifuged at 10'000 rpm for 10 min at 4°C. The same extraction procedure was used for total GGT activity, but using the infiltration buffer.

### ASC and DHA determination

Ascorbate and dehydroascorbate were measured by spectrophotometric analysis following the decrease in absorbance at 265 nm according to Hewitt and Dickes (1961).

### Chromatographic low-molecular-weight thiol assay

To measure total thiol concentration extracts, 50 μL of total leaf extract and ECWF were derivatized with 4-fluoro-7-sulfobenzofurazan ammonium salt fluorophore (SBD-F) (Dojindo, Japan). Low Molecular Weight (LMW) thiols were separated by isocratic High pressure liquid chromatography (HPLC) using the method described elsewhere (Masi et al., 2002) with some modifications. The mobile phase was 75 mM ammonium-formiate, pH 2.9 and 3% methanol (97:3, vol/vol). For oxidized thiol quantification, samples were pre-treated with 2-vinylpyridine according to Griffith (1980), then buffered to basic pH and treated with 2-vinylpyridine for 1 h to protect the free thiol moieties. Afterwards, the samples were washed to remove the resulting complexes, and the remaining unreacted samples (containing the oxidized thiols) were derivatized and analyzed by HPLC.

### GGT activity measurements

GGT activity was determined spectrophotometrically according to Huseby and Stromme (1974). Leaf extracts were reacted in a mix of solution A (5 mM g-glutamyl-p-nitroanilide 100 mM NaH_2_PO_4_, pH 8.0) and solution B (575 mM gly-gly in 100 mM NaH_2_PO_4_, pH 8.0) in a ratio of 10:1. Absorbance was recorded for 1 h at 407 nm to measure p-nitroaniline release into the assay medium.

### Statistical analysis

After checking for a normal distribution, data were tested with One-Way ANOVA using the General linear models (GLM) procedure in SAS (SAS 9.2, 2008). Data with a non-normal distribution were submitted to a non-parametric test (Kruskal-Wallis) using XLSTAT (2014 version). In both cases, Bonferroni's test was used to ascertain differences between means. Significance was established at *P* = 0.05.

### Proteomic analysis

#### Protein *in situ* digestion

Proteins obtained from infiltration were quantified by bicinchoninic acid spectrophotometric assay; 50 μg of proteins were loaded in a homemade 11% Sodium dodecyl sulfate (SDS) gel and the electrophoretic run was stopped as soon as the protein extracts entered the running gel. Bands were excised and washed several times with 50 mM triethylammonium bicarbonate (TEAB) and dried under vacuum after a short acetonitrile wash. Cysteines were reduced with 10 mM dithiothreitol (in 50 mM TEAB) for 1 h at 56°C, and alkylated with 55 mM iodoacetamide (in 50 mM TEAB) for 45 min at room temperature in the dark. Gel pieces were then washed with alternate steps of TEAB and acetonitrile, and dried. Proteins were digested *in situ* with sequencing grade modified trypsin (Promega, Madison, WI, USA) at 37°C overnight (12.5 ng·μl^−1^ trypsin in 50 mM TEAB). Peptides were extracted with three steps of 50% acetonitrile in water. One μg of each sample was withdrawn to check digestion efficiency using LC-MS/MS analysis, and the remaining peptide solution was dried under vacuum.

### iTRAQ labeling and peptide fractionation

Peptides were labeled with iTRAQ reagents (ABSciex) according to the manufacturer's instructions. They were labeled with the four iTRAQ tags using a Latin panel strategy: wt UV-B, *ggt1* UV-B, wt ctrl, and *ggt1* ctrl were labeled respectively with 114, 115, 116, and 117 tags in the first replicate; 115, 116, 117, 114 tags in the second and 116, 117, 114, 115 tags in the third. Prior to mixing the samples in a 1:1:1:1 ratio, 1 μg of each sample was analyzed separately to check label efficiency by LC-MS/MS analysis, setting the iTRAQ labeling as a variable modification in the database search. All the peptides were correctly identified as being iTRAQ-modified at the N-terminus and at each lysine residue. The samples were then pooled and dried under vacuum. The mixture of labeled samples (one per replicate) was suspended in 500 μl of buffer A (10 mM KH_2_PO_4_, 25% acetonitrile, pH 2.9) and loaded onto a strong cation exchange cartridge (AB Sciex) for fractionation according to (Tolin et al., 2013). After a washing step with buffer A, the peptides were eluted stepwise with increasing concentrations of KCl in buffer A (25, 50, 100, 200, and 350 mM). The volume of each fraction (500 μl) was reduced under vacuum, and the samples were desalted using C18 cartridges (Sep-Pack, C18, Waters) according to the manufacturer's instructions. The samples were ultimately dried under vacuum and kept at −20°C until MS analysis.

### LC-MS/MS analysis, database search, and protein quantification

Samples were suspended in H_2_O/0.1% formic acid and analyzed by LC-MS/MS. The MS analyses were conducted with a LTQ-Orbitrap XL mass spectrometer (Thermo Fisher Scientific, Pittsburgh, CA, USA) coupled online with a nano-HPLC Ultimate 3000 (Dionex - Thermo Fisher Scientific). Samples were loaded in a homemade 10 cm chromatographic column packed into a pico-frit (75 mm id, 10 mm tip, New Objectives) with C18 material (ReproSil, 300 Å, 3 μm). The LC separation and mass spectrometer settings used for the analyses were the same as those described in Tolin et al. (2013), and the method was as described by Köcher et al. (2009).

The raw LC-MS/MS files were analyzed using Proteome Discoverer 1.4 (Thermo Fisher Scientific), connected to a Mascot Search Engine server (version 2.2.4, Matrix Science, London, UK). The spectra were searched against a ARATH Uniprot protein database. Enzyme specificity was set to trypsin with two missed cleavages, and peptide and fragment tolerance was set to 10 ppm and 0.6 Da, respectively. Methylthiocysteine, 4-plex iTRAQ at the N-terminus and Lys were set as fixed modifications, while Met oxidation was selected as a variable modification. False discovery rates (FDR) were calculated by the software, based on the search against the corresponding randomized database. Only proteins identified and quantified with at least 2 unique peptides with 99% confidence (FDR 1%) were considered as positive identifications. A 5% FDR was adopted in only two cases (as shown in **Table 2** and in Data Sheet 1, Supplementary Material), in which the MS/MS spectra were manually inspected for confirmation. Data were pre-filtered to exclude MS/MS spectra containing less than 5 peaks or with a total ion count below 50. Quantification was done by normalizing the results on the median value of all measured iTRAQ reporter ratios.

Protein expression ratios are given as: wt (UV-B/ctrl), *ggt1* (UV-B/ctrl), ctrl (*ggt1*/wt), and UV-B (*ggt1*/wt) and they are the mean value of at least 2 biological replicates (see Data Sheet 2, 3, 4, Supplementary Material). To improve the statistical robustness of the data, all proteins were submitted to a two-tailed *Z* test with a confidence level of *p* < 0.05. The variations were further restricted to proteins exhibiting an at least ±50% fold change in their expression (1.5 for upregulated and 0.68 for downregulated proteins).

## Results

### GGT activity

**Figure 1:**
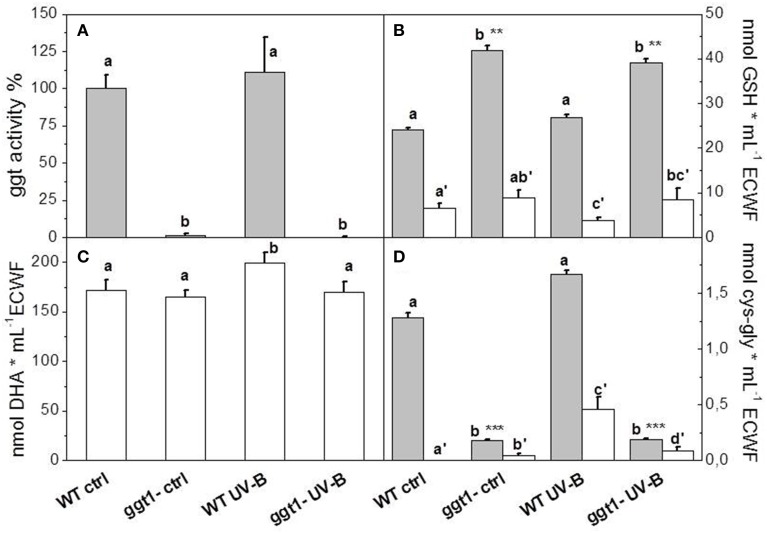
**GGT activity (A), glutathione (B), ascorbate (C) and cys-gly (D) in ECWF**. Gray bars show total content, white bars oxidized forms. Reported values are the mean ± S.E. of 3 technical replicates, each conducted with at least 4 biological replicates. Different letters indicate significant differences between conditions (*P* = 0.05^*^; *P* = 0.01^**^; *P* = 0.001^***^). For GGT activity, the reference value of the wild type control was 43.05 mU/mL ECWF.

An increase in GGT enzymatic activity was found in wt plants after UV-B irradiation; this increase was greater in total leaf extracts (+35%, Table 1) than in ECWF (+10%, Figure 1A). Activity in the mutant was significantly lower in total leaf extracts and almost undetectable in the ECWF (as was to be expected because GGT1 is the only apoplastic isoform active in leaves), but no significant differences were observed after UV-B exposure (Figure 1A).

**Table 1:** **GGT activity, ascorbate, GSH and cys-gly content in total leaf extract**.

	**ggt activity %**	**Ascorbate μmol/ g FW**	**GSH nmol/g FW**	**cys-gly nmol/g FW**
wt ctrl	100 ± 7.8	2.82 ± 0.03	261.9 ± 5.8	1.14 ± 0.04
*ggt1* ctrl	5 ± 1.6	2.66 ± 0.03	275.7 ± 7.5	1.04 ± 0.04
wt UV-B	136 ± 11	3.37 ± 0.05^*^	296.2 ± 6.2	1.34 ± 0.05
*ggt1* UV-B	7 ± 3	3.53 ± 0.06^*^	293.7 ± 9.1	1.01 ± 0.03

*Values are the mean ± S.E. of 4 biological replicates from 3 technical replicates. For GGT activity, the reference value of the wild type control was 50.43 mU/g FW. Asterisks indicate P ≤ 0.05*.

### Antioxidant content (GSH and ascorbate)

Ascorbate was only found in its reduced form in total leaf extracts, and was increased by UV-B treatment (by approximately 20–30%) in both genotypes (Table 1). We found no reduction in the ascorbate in the apoplastic space, where we could only measure the oxidized form, dehydroascorbate (Figure 1C). We found no significant differences between the genotypes or treatments in the total glutathione or cys-gly content in total leaf extract (Table 1). In ECWF total glutathione content was higher in the *ggt1* mutant than in the wild type; and supplementing UV-B radiation did not alter these values (Figure 1B). GSSG was lower in the ECWF from wt leaves under UV-B treatment, whereas oxidized cys-gly increased significantly under the same conditions (Figures 1B,D).

It should be noted that apoplastic glutathione is only a small fraction of total leaf glutathione, so fluctuations in the apoplast are somewhat diluted during the extraction process. For the same reason, variations in the small amount of extracellular DHA may not have been reflected in total leaf extracts.

### Proteomic analysis

In total, 329 proteins were uniquely identified by the LC-MS/MS analyses; 208 were found in at least two biological replicates. Based on the Gene Ontology (GO) assignment for cellular compartmentalization (Uniprot 14, www.uniprot.org), we restricted our analysis to the 118 proteins that were either apoplastic or unlocalized, accounting for approximately 57% of the total.

Should be considered bearing in mind that several truly extracellular proteins have yet to be properly assigned to the apoplast. In fact, it has been reported (Agrawal et al., 2010; Ding et al., 2012) that about 50% of proteins secreted in the apoplast lack a leaderless secretory tag;. There are consequently many unpredicted secretory proteins in plants, and their occurrence is often underestimated or they are even considered improperly as contaminants. Our decision to restrict our assignments according to the Uniprot database was therefore rigorous, but probably led to an underestimation of the truly apoplastic proteins.

The variations considered were further restricted to proteins exhibiting an at least ±50% fold change in expression.

Various information can be drawn from comparisons between the four experimental conditions: (i) the effect of UV-B treatment on each genotype; (ii) differential apoplastic protein composition in *ggt1* vs. wt; (iii) possible differences in the behavior of the *ggt1* mutant and the wt under UV-B. Comparing the two genotypes, 23 proteins were downregulated and only three were upregulated in *ggt1* by comparison with the wt under physiological conditions (Table 2 and Data Sheet 1, Supplementary Material). UV-B treatment resulted in 8 proteins being downregulated in *ggt1*; and in 12 being downregulated and 11 being upregulated in the wt. When the *ggt1* and wt were compared after UV-B treatment, it emerged that 9 proteins were expressed less, and 10 were expressed more in the mutant than in the wild type. A condensed view of all these variations is given in Table 2.

**Table 2:** **Brief overview of expression changes in apoplastic and unlocalized proteins in the four conditions analyzed: wt (UV-B/ctrl), *ggt1* (UV-B/ctrl), ctrl (*ggt1*/wt), and UV-B (*ggt1*/wt)**.

**Accession nr**	**Locus name**	**Description**	**FDR %**	***WT* UVB/ctrl**	***ggt1* UVB/ctrl**	**CTRL *ggt1/wt***	**UV-B *ggt1/wt***
F4HR88	At1g33590	Leucine-rich repeat-containing protein	1	0.55		0.48	
O81862	At4g19810	At4g19810	1	0.55			
F4IAX0	At1g31690	Putative copper amine oxidase	1	0.57			
Q9M5J8	At5g06870	Polygalacturonase inhibitor 2	1	0.57			
Q9LMU2	At1g17860	At1g17860/F2H15_8	1	0.57		0.48	
B9DGL8	At5g08370	AT5G08370 protein	1	0.58			
F4HSQ4	At1g20160	Subtilisin-like serine endopeptidase-like protein	1	0.61			
F4IIQ3	At2g28470	Beta-galactosidase	1	0.62			
Q9ZVS4	At1g03220	Aspartyl protease-like protein	1	0.65		0.66	2.5
Q94F20	At5g25460	At5g25460	1	0.66		0.58	1.6
Q9FT97	At5g08380	Alpha-galactosidase 1	1	0.68			
Q940J8	At4g19410	Pectinacetylesterase family protein	1	0.68		0.68	1.9
O49006	At3g14310	Pectinesterase/pectinesterase inhibitor 3	1	1.5			0.55
O65469	At4g23170	Putative cysteine-rich receptor-like protein kinase 9	1	1.5			
P24806	At4g30270	Xyloglucan endotransglucosylase/hydrolase prot 24	1	1.6			
F4J270	At5g20950	Beta-1,3-glucanase 3	1	1.7			0.47
Q9ZV52	At2g18660	EG45-like domain containing protein 2	1	1.8			
P46422	At4g02520	Glutathione S-transferase F2	1	1.8			0.51
O22126	At2g45470	Fasciclin-like arabinogalactan protein 8	1	1.9			
F4JRV2	At4g25100	Superoxide dismutase	5	1.9		1.7	
P33157	At3g57260	Glucan endo-1,3-beta-glucosidase, acidic isoform	1	2.1	0.63		0.26
F4JBY2	At3g60750	Transketolase	1	2.7		2.2	
O80852-2	At2g30860	Isoform 2 of Glutathione S-transferase F9	1	2.9			
F4HUA0	At1g07930	Elongation factor 1-alpha	1	4.4			
Q9SG80	At3g10740	Alpha-L-arabinofuranosidase 1	1		0.35		
Q9FZ27	At1g02335	Germin-like protein subfamily 2 member 2	5		0.37		
F4K5B9	At5g07030	Aspartyl protease family protein	1		0.54		
O64757	At2g34930	Disease resistance-like protein/LRR domain-containing protein	1		0.31		
Q9S7Y7	At1g68560	Alpha-xylosidase 1	1		0.55		
Q9C5C2	At5g25980	Myrosinase 2	1		0.61		
Q9FKU8	At5g44400	Berberine bridge enzyme	1		0.50		0.68
Q9SMU8	At3g49120	Peroxidase 34	1			0.56	
Q9ZVA2	At1g78830	At1g78830/F9K20_12	1			0.57	2.3
P94072	At5g20630	Germin-like protein subfamily 3 member 3	1			0.52	
Q42589	At2g38540	Non-specific lipid-transfer protein 1	1			0.42	
Q9FW48	At1g33600	Leucine-rich repeat-containing protein	1			0.58	
Q9LXU5	At5g12940	Leucine-rich repeat-containing protein	1			0.51	
Q9LYE7	At5g11420	Putative uncharacterized protein At5g11420	1			0.55	
Q9M2U7	At3g54400	AT3g54400/T12E18_90	1			0.64	2.0
Q9LT39	At3g20820	Leucine-rich repeat-containing protein	1			0.68	
O24603	At2g43570	Chitinase class 4-like protein	1			0.34	0.17
P33154	At2g14610	Pathogenesis-related protein 1	1			0.34	
Q8W112	At5g20950	Beta-D-glucan exohydrolase-like protein	1			0.65	
P28493	At1g75040	Pathogenesis-related protein 5	1			0.30	
Q94K76	At5g18470	Curculin-like (Mannose-binding) lectin family protein	1			0.53	
Q9LEW3	At5g10760	Aspartyl protease family protein	1			0.44	
Q9LRJ9	At3g22060	Cysteine-rich repeat secretory protein 38	1			0.49	
Q9LV60	At5g48540	Cysteine-rich repeat secretory protein 55	1			0.5	
Q9C5M8	At4g24780	Probable pectate lyase 18	1			0.68	
O23255	At4g13940	Adenosylhomocysteinase 1	1			1.5	
O50008	At5g17920	5-methyltetrahydropteroyltriglutamate-homocysteine methyltransferase	1			2.4	
Q9SVG4-2	At4g20830	Isoform 2 of Reticuline oxidase-like protein	1				0.43
Q940G5	At4g25900	Aldose 1-epimerase family protein	1				0.61
Q9LFA6	At3g52840	Beta-galactosidase 2	1				0.59
Q9LU14	At3g16370	GDSL esterase/lipase APG	1				1.6
Q39099	At2g06850	Xyloglucan endotransglucosylase/hydrolase prot 4	1				1.8
Q9LFR3	At5g14920	Gibberellin-regulated protein 14	1				1.8
O04496	At1g09750	Aspartyl protease-like protein	1				1.9
Q9FH82	At5g45280	AT5g45280/K9E15_6	1				2.0

**Figure 2:**
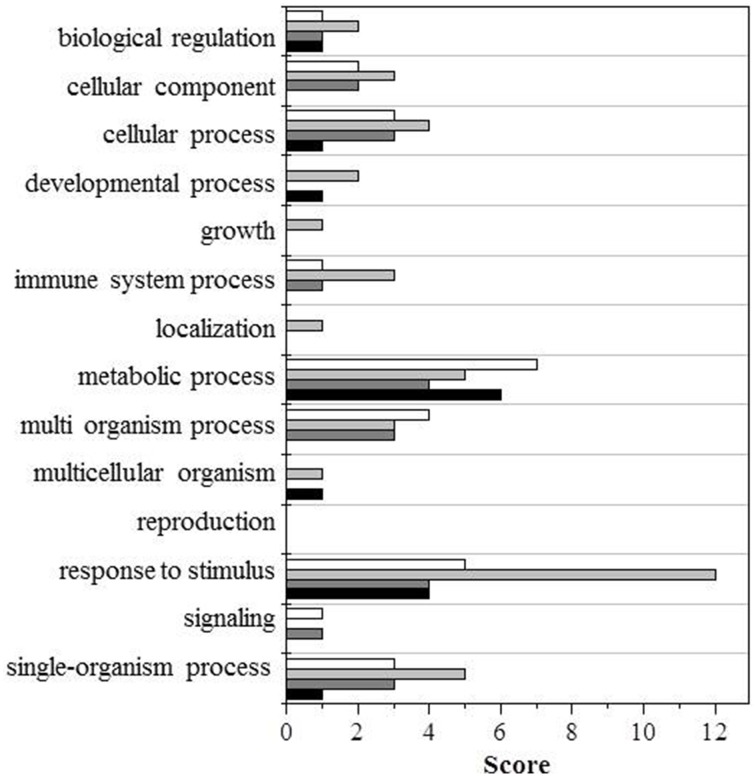
**GO terms distribution in the biological process of downregulated proteins**. Black bars shows wt (UV-B/ctrl), dark gray bars *ggt1* (UV-B/ ctrl), light gray is ctrl (*ggt1*/wt), and white bars is UV-B (*ggt1*/wt).

**Figure 3:**
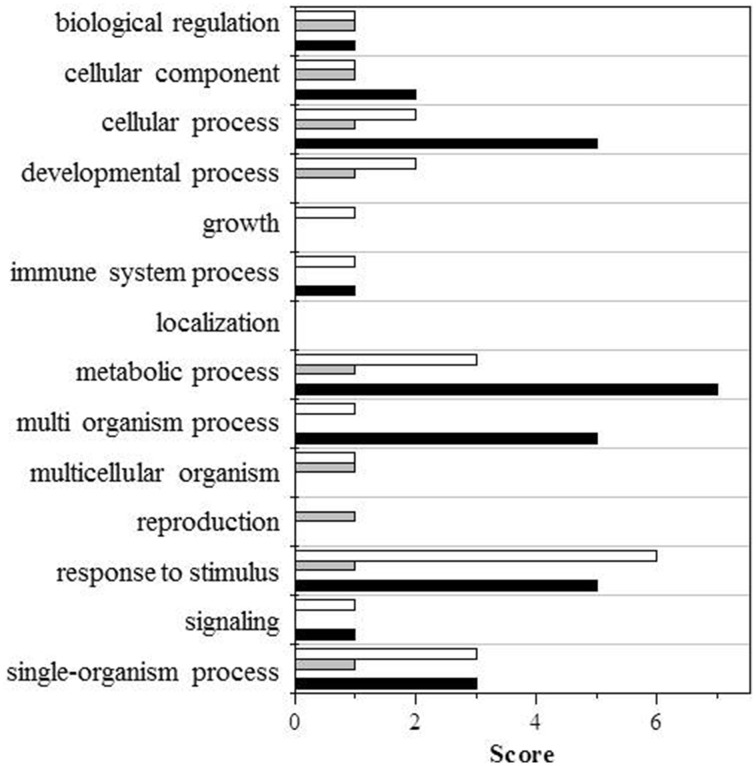
**GO terms distribution in the biological process of upregulated proteins**. Black bars shows wt (UV-B/ctrl), dark gray bars *ggt1* (UV-B/ ctrl), light gray is ctrl (*ggt1*/wt), and white bars is UV-B (*ggt1*/wt).

To facilitate the interpretation of the results, we ran a bioinformatic analysis with Blast2GO, a tool for the functional annotation of sequences and data mining, based on the GO vocabulary (Conesa and Götz, 2008). This made it easy to assess and visualize the relative abundance of functional terms (obtained from the pool of GO terms) in the category of biological processes, based on a score assigned by the Blast2GO algorithm. Within the category of biological processes, the GO terms involved under the four conditions, and either down- or upregulated, are shown in Figures 2, 3, respectively.

Based on the Blast2GO scores, UV-B in both the wild type and the *ggt1* mutant mainly seem to cause a lower expression of proteins in the “metabolic process” and “response to stimulus” categories (Figure 2). Far fewer proteins were upregulated, but the analysis as a whole again showed that the “response to stimulus” and “metabolic process” categories scored highest, but only after the UV-B treatment in both genotypes (Figure 3). Based on the results shown in Table 2, the variations observed were functionally grouped as explained below. For the sake of simplicity, the proteins listed in Table 2 were divided into 4 broad categories, but many of those described here could have been placed in more than one category (depending on whether we considered the protein's biological function or its catalytic activity, for instance).

### Pathogenesis and hormone-related proteins

Gibberellins are hormones that can be found in the apoplastic space too (Kramer, 2006). Here, we found the gibberellin-regulated protein Q9LFR3 (At5g14920) upregulated by UV-B treatment in the mutant.

Among the proteins targeted by hormones there is a galactose-binding domain containing protein (At5g25460, Q94F20) with a putative function in response to karrikins, a novel group of plant growth regulators (Nelson et al., 2011). This protein is downregulated in the wild type under UV-B treatment, and in the *ggt1* mutant in physiological conditions.

By comparison with the wild type, two pathogen-related proteins are less expressed in the *ggt1* mutant, i.e., PR-1 (At2g14610) and PR-5 (At1g75040), reportedly regulated by brassinosteroids (Sävenstrand et al., 2004). Another protein involved in lipid catabolism and response to pathogens is a GDSL esterase/lipase (At3g16370) that is expressed more in the *ggt1* mutant than in the wild type under UV-B.

Proteolytic enzymes are directly or indirectly involved in several plant cellular processes, including resistance to pathogens and disease (Xia et al., 2004). In our study, we identified four members of the aspartyl protease family, a class of enzymes acting as endopeptidases to remove aspartic residues from polypeptide chains. One of them (At1g03220) is downregulated in the wild type after UV-B treatment, and in the *ggt1* mutant in physiological conditions. This protein and At1g09750 are both upregulated in *ggt1* by comparison with the wild type as an effect of UV-B treatment. At5g07030 is downregulated under UV-B in the mutant, and At5g10760 is downregulated in the mutant under control conditions. It could be hard to explain these opposite effects in the expression of members of the same aspartyl protease family, but it is worth noting that these enzymes are reportedly involved in plant defenses and development (Minic et al., 2007).

### Cell wall remodeling

Up to 90% of plant cell walls consist of three types of polysaccharide: cellulose, hemicelluloses, and pectins. Their composition and structure differ from one species to another, and change as plants develop and with environmental fluctuations (Cosgrove, 1997; Popper and Fry, 2003; Minic et al., 2007). Six proteins belonging to the class of glycoside hydrolases are downregulated by UV-B radiation in the wild type (At2g28470, At5g08380, At4g19410, At5g06870, At4g19810, and At5g08370) while another six proteins are upregulated (At3g14310, At2g45470, At3g57260, At4g30270, At5g20950, and At2g18660).

Comparing the *ggt1* mutant with the wild type clearly revealed a constitutive downregulation of proteins related to cell wall remodeling (At4g19410, At4g24780, At5g20950, and At2g43570). One of them is downregulated both in the mutant under physiological conditions and in the wild type after UV-B treatment. An opposite response to UV-B radiation emerged for beta-glucosidase At3g57260, which was higher in the wild type, and lower in *ggt1* after the treatment. UV-B radiation resulted in a lower expression in the *ggt1* mutant of other cell wall remodeling proteins, namely α-arabinofuranosidase At3g10740, α-xylosidase At1g68560, and the berberine bridge enzyme At5g44400. We also found a lower expression of chitinase At2g43570 in the *ggt1* mutant than in the wild type both under physiological conditions and after treatment with UV-B.

### Signaling

In this study, we observed changes in four proteins containing leucine-rich repeats, and in two cysteine-rich secretory proteins belonging to a class acting as kinases. Leucine- and cysteine-rich proteins are transmembrane proteins that are reportedly induced by ROS and salicylic acid (Brandes et al., 2009).

All leucine-rich proteins were downregulated in the mutant (At1g33600, At5g12940, At1g33590, and At3g20820) under physiological conditions. It seems particularly interesting that At1g33590 expression was also downregulated in the wild type under UV-B radiation.

The two cysteine-rich repeat secretory proteins, At3g22060 and At5g48540, were both downregulated in the *ggt1* mutant under physiological conditions. These proteins are also PM-associated receptor-like kinases, and At3g22060 interacts with one or more unknown PM-localized ABA receptor(s) (Xin et al., 2005), whereas At5g48540 is involved in response to karrikins (Nelson et al., 2011).

The EG45-like domain containing protein (At2g18660) is part of a class of small proteins that act as signaling molecules. In our study, At2g18660 was upregulated in the wild type under UV-B.

### GSTs, redox regulation and ROS balance

One protein (At1g31690) involved in response to oxidative stress was downregulated under UV-B in the wild type. This protein is involved in H_2_O_2_ metabolism, acting as an oxidase. Also Peroxidase 34 (At3g49120) was downregulated in the *ggt1* mutant under control conditions.

Two Glutathione S-Transferases (GSTs) proteins belonging to categories F2 and F9 (At4g02520 and At2g30860, respectively) were upregulated in the wild type plants after UV-B radiation. When the two genotypes were compared after the same treatment, the expression level was lower in the former.

Two proteins in the germin-like family (At5g20630 and At1g02335) were downregulated in *ggt1*, one in physiological conditions and the other after UV-B treatment. These proteins are involved in defending against biotic and environmental stress.

Superoxide dismutase proteins are reportedly involved in removing superoxide radicals from the apoplast following UV-B radiation (Alscher et al., 2002). In our study, the expression of superoxide dismutase (At4g25100) was upregulated in the wild type after UV-B radiation and in *ggt1* under physiological conditions.

## Discussion

### GGT activity and soluble antioxidants

Following excess UV-B exposure, plants deploy a wide array of morphological and biochemical defense mechanisms, including soluble antioxidants (Shiu, 2005). The changes observed in this study are consistent with the view that, under UV-B radiation, oxidative conditions in the apoplastic space involve both ascorbate and glutathione, the two main soluble antioxidant molecules in plant cells. Ascorbate in ECWF was found fully oxidized, which is consistent with the view that oxidizing conditions prevail in the apoplastic space (Vanacker et al., 1998a,b; Saruhan et al., 2009).

UV-B radiation induced an increase in apoplastic ascorbate in both genotypes, suggesting that ascorbate is extruded as a means to counteract the artificially-imposed oxidative conditions. While glutathione content was substantially unchanged in total leaf extracts in all the conditions tested, it was altered in the ECWF from mutant leaves, where the effect of the *ggt1* null mutation results in a net increase in glutathione content, as a predictable effect of the reduced GGT degradation activity.

Under UV-B, the concurrent decrease in oxidized glutathione and increase in oxidized cys-gly can be interpreted as an enhanced gamma-glutamyl transferase activity; this is supported by the previous finding that GGT1 has a stronger preference for GSSG (Ohkama-Ohtsu et al., 2008).

GGT activity was barely detectable in the mutant, confirming that GGT1 is the main isoform contributing to over 90% of said activity in wild type leaves. Since the GGT2 isoform is not expressed in leaves (Destro et al., 2011) and GGT3 is assumed to be non-functional (Martin et al., 2007), this indicates that the activity found in the mutant represents the contribution of the remaining vacuolar isoform GGT4.

The increased GGT activity following UV-B treatment in the wild type therefore suggests that the rate of the gamma-glutamyl cycle is accelerated by this radiation. The involvement of the vacuolar GGT4 in the degradation of glutathione conjugates, e.g., with lipoperoxides and/or other damaged molecules, might be implicated too, but this seems unlikely since no significant increase in GGT activity was apparent in the mutant under the same conditions.

Collectively, these novel findings thus imply that the gamma-glutamyl cycle is accelerated under oxidative conditions imposed by ultraviolet-B radiation, and support the conviction that it is involved in oxidative stress sensing and/or response.

### Apoplastic proteome readjustments

Proteome analysis has proved a powerful tool for deciphering cell metabolism under different perturbations and has been found useful in apoplastic studies too (Agrawal et al., 2010). Apoplastic proteins establish a constitutive systemic defense network, with only a few of them changing under environmental and/or biotic stress (Delaunois et al., 2014).

Two main approaches are currently adopted in plant physiology studies: the application of stress conditions, and the use of mutants. These alternative and converging strategies may provide tools for deciphering metabolism. In this work, oxidative conditions were imposed with UV-B and studied in redox-altered *ggt1* mutants. Subcellular fractionation and apoplastic proteome analysis were then used to arrive at a better understanding of the rearrangements in the extracellular compartment.

The experimental design adopted here could consequently help to describe and compare the effects of UV-B treatment on the two genotypes, and the differences in apoplastic proteome composition between the mutant and wild type leaves under control conditions.

In both genotypes, UV-B treatment caused a downregulation of different kinds of protein related to cell wall biosynthesis, response to stress and proteolysis. It prompted an upregulation, but only in the wild type, of other proteins involved in cell wall remodeling and two glutathione S-transferases, GST-F2 and GST-F9. No proteins were found upregulated in the *ggt1* mutant after UV-B (Table 2).

The hormonal changes occurring in the *ggt1* mutant, with or without exposure to UV-B radiation, were not considered in the experimental setup, and were beyond the scope of this work. Several proteins seen here to change in expression could be targets for hormones, however. For instance, one protein whose expression was stimulated by UV-B is reportedly a gibberellin-regulated protein (At5g14920), suggesting that gibberellins could be implicated in the response. The expression of a galactose-binding domain containing protein (At5g25460), which is stimulated by karrikins (a novel group of plant growth regulators (Nelson et al., 2011), was also higher in the mutant than in the wild type after UV-B. Interplay with hormones may also concern two PRPs (PR-1 and PR-5), whose expression was lower in the *ggt1* mutant under physiological conditions. A previous study (Sävenstrand et al., 2004) had found their expression strongly reduced in brassinosteroid metabolism mutants. It would be interesting to see whether the brassinosteroid pathway is altered in *ggt1* mutants too.

Broadly speaking, cell wall modifying proteins such as glycosyl hydrolases (GHs), peroxidases, esterases, transglycosylases, and lyases, are involved in the construction, remodeling or turnover of cell wall components (Cosgrove, 1997; Stolle-Smits et al., 1999; Obel et al., 2002; Reiter, 2002). Some of them may have other functions too, e.g., in the glycosylation state of target proteins (Kang et al., 2008), which in turn could be involved in signaling processes (Minic et al., 2007).

Taking a broader look at the changes found in this category suggests that UV-B affects the expression of some proteins in the wild type (e.g., pectine-acetylesterase and its inhibitor, xyloglucan endotransglucosylase, beta 1,3-glucanase, beta-galactosidase, alpha-galactosidase and a polygalacturonase inhibitor) and others in the *ggt1* mutant (alpha-xylosidase, myrosinase, alpha-arabinofuranosidase, and a berberine bridge enzyme), confirming the view that the cell walls are the target of this radiation. Notably, these remodeling processes are affected in the *ggt1* mutant not only by UV-B treatment, but also under physiological conditions. Since cell wall structure is reportedly altered during development and by exposure to stress (Potters et al., 2009), our findings could be explained by the existence of a stress-like condition in the mutant, where some signals mimic the oxidative state induced by UV-B in the wild type.

Myrosinase, a protein in the class of glycoside hydrolases, was less expressed under UV-B in the *ggt1* mutant, and this could have ecophysiological consequences because in Brassicaceae myrosinases play a part in growth, development, and defenses against microbes, as well as deterring insects and herbivores (Rodman, 1991). The two germin-like proteins that were downregulated in the *ggt1* mutant could also be consistent with alterations in the defense systems against biotic and environmental stress.

The expression of some other proteins was altered in opposite ways (up- or downregulated) after UV-B exposure, depending on the genotype considered: for instance, the stress-responsive glucan endo-1,3-beta-glucosidase was upregulated in the wild type, but downregulated in *ggt1*.

A group of proteins involved in response to stimuli, i.e., the leucine-rich and cysteine-rich proteins, was downregulated in *ggt1* in physiological conditions. Leucine-rich proteins contain a leucine-rich repeat (LRR) motif that has revealed a central role in recognizing different pathogen-associated molecules in the innate host defense of plants and animals (Gunawardena et al., 2011).

In this study, we also identified 4 aspartyl proteases that were altered under our experimental conditions: this may mean that members of this category of enzymes related to plant defenses are sensitive to redox variations. Aspartyl proteases are important for plant development. They have been implicated in the ABA-dependent responsiveness to drought-induced stress (Yao et al., 2012), and in Arabidopsis a gene encoding the aspartyl protease protein was found downregulated by cold and high-salinity stress (Seki et al., 2002).

The EG45-like domain containing protein 2 (At2g18660) was upregulated in the wild type under UV-B. This protein belongs to the category of plant natriuretic peptides (PNPs), a novel class of small proteins showing homology with the N-terminus of expansins, though they are significantly shorter and lack the wall-binding domain (Ludidi et al., 2002). Previous studies found PNPs upregulated under saline and osmotic conditions (Rafudeen et al., 2003), but the effects of UV-B on this class of peptidic signaling molecules had not been reported before.

Among the variations in apoplastic enzyme expression found in the present study, some that particularly attracted our attention are closely related to ROS metabolism.

Superoxide anion formation is reportedly triggered by ultraviolet-B radiation (Alscher et al., 2002). It seems noteworthy that no plant superoxide dismutase (SOD) identified to date contains a signal peptide, but extracellular SOD activity in stressed or pathogen-infected plants has been reported in many works (Hernandez et al., 2001; Karpinska et al., 2001; Kaffarnik et al., 2009; Pechanova et al., 2010). SODs produce H2O2, which is degraded to H2O by ascorbate peroxidase. By removing superoxide anions, SODs may limit the duration of the oxidative burst to an early event in plant defense (Pristov et al., 2013; Scheler et al., 2013).

In this study, a superoxide dismutase (At4g25100) was found upregulated by UV-B radiation in wild type leaves: this can be interpreted as the need to improve scavenging activity to remove excess superoxide anions. Although its localization is not reported in the official databases, its occurrence in the apoplast was noted in other studies too (Kwon et al., 2005; Ding et al., 2012). Higher SOD levels combined with lower levels of the putative copper amine oxidase (At1g31690) may result in higher H_2_O_2_ levels. An increased GST expression could also result in its scavenging, however, so while it seems clear that ROS metabolism is affected by UV-B treatment in the wild type, it is hard to draw any conclusions on H_2_O_2_ levels, and further experimentation is needed to validate our hypothesis.

The ROS scavenging scenario in the *ggt1* mutant is undoubtedly more complex. The above-mentioned apoplastic SOD is upregulated under physiological conditions, and a peroxidase is downregulated. These effects may be interpreted as readjustments in the redox-altered *ggt1* background. Such readjustments may be needed to sustain a higher H_2_O_2_ level, which could act as a signal.

**Figure 4:**
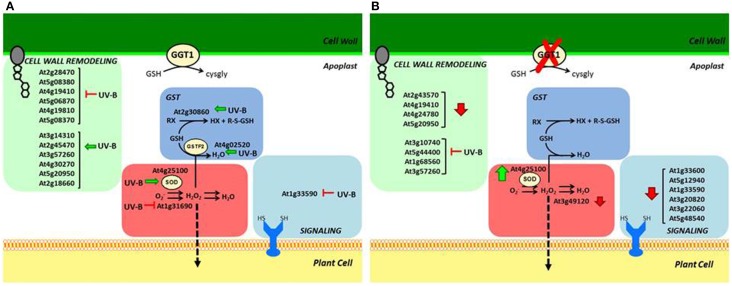
**Schematic overview of apoplastic proteome variations in: (A) wild type, induced by UV-B; (B) *ggt1* genotype due to the mutation and/or to UV-B treatment**. Vertical arrows refer to stimulation (↑) or repression (↓) caused by the mutation; horizontal arrows indicate repression (⊢) or stimulation (←) caused by UV-B treatment.

Taken together, these effects may result in higher H_2_O_2_ levels in the mutant under physiological conditions (schematically shown in Figure 4), whereas the rise in H_2_O_2_ in the wild type is a direct consequence of oxidative stress conditions induced by UV-B radiation.

As a signaling molecule, H_2_O_2_ may cross membranes in a process facilitated by aquaporins (Bienert et al., 2006), reaching internal cell compartments and the nucleus, where it can activate defense gene expression (Mullineaux et al., 2006). If this assumption holds true, it might explain the “constitutive alert response” effect observed in a previous proteomic analysis of total leaf extracts from *ggt1* mutant leaves (Tolin et al., 2013).

Future research is therefore needed to ascertain the level of ROS, and especially H_2_O_2_, in the apoplast of *ggt1* mutants, and the possible involvement of hormones (e.g., brassinosteroids and gibberellins) in the response. Both H_2_O_2_ and hormones are signals arising in the apoplast that can be transferred intracellularly and evoke the cell's responses. For this signal transduction function we could also consider four leucine-rich and two cysteine-rich proteins belonging to the superfamily of receptor-like kinases (RLKs), which are associated with the plasma membrane and contain redox-sensitive thiols, which were found at lower level in the ggt1 mutant. Disrupting of the gamma-glutamyl cycle could result in an altered signal perception pathway.

While hormonal and redox readjustments seem to be implicated in the modified metabolism of *ggt1* mutants, it remains to be seen how silencing the gamma-glutamyl transferase activity and consequently impairing the gamma-glutamyl cycle may lead to the effects reported here. Further experiments are needed to clarify the link between the gamma-glutamyl cycle and apoplastic redox events.

### Conflict of interest statement

The authors declare that the research was conducted in the absence of any commercial or financial relationships that could be construed as a potential conflict of interest.

## Supplementary material

The mass spectrometry proteomics data have been deposited to the ProteomeXchange Consortium via the PRIDE partner repository with the dataset identifier PXD001807.

## Abbreviations

UV-B: Ultraviolet-B radiation
ROS: Reactive Oxygen Species
GGT1: gamma-glutamyl transferase 1 isoform
H_2_O_2_: Hydrogen Peroxide
iTRAQ: Isobaric tags for relative and absolute quantification
UVR8: UV-B photoreceptor 8
GSH: Glutathione
LC-MS-MS: Liquid Chromatography Mass Spectrometry
ECWF: Extracellular washing fluid
DHA: Dehydroascorbate
SBD-F: 4-fluoro-7-sulfobenzofurazan ammonium salt fluorophore
LMW: Low Molecular Weight
HPLC: High pressure liquid chromatography
ANOVA: Analysis of variance
GLM: General linear models
SDS: Sodium dodecyl sulfate
TEAB: Triethyl ammonium bicarbonate
FDR: False discovery rates
FW: Formula Weight
GSSG: Glutathione disulfide/Oxidized GSH
SOD: Superoxide dismutase
PNPs: Plant natriuretic peptides
GSTs: Glutathione S-Transferases
GHs: Glycosyl hydrolases
PRPs: Pathogenesis-related proteins.
